# Cancer of the vagina: 2021 update

**DOI:** 10.1002/ijgo.13867

**Published:** 2021-10-20

**Authors:** Tracey S. Adams, Linda J. Rogers, Mauricio A. Cuello

**Affiliations:** ^1^ Department of Gynecological Oncology Groote Schuur Hospital University of Cape Town Cape Town South Africa; ^2^ South African Medical Research Council University of Cape Town Gynecological Cancer Research Centre (SA MRC UCT GCRC) Cape Town South Africa; ^3^ University of Cape Town Global Surgery Cape Town South Africa; ^4^ Division of Obstetrics and Gynecology School of Medicine Pontificia Universidad Católica de Chile Santiago Chile

**Keywords:** adenocarcinoma, FIGO Cancer Report, HPV, imaging techniques, individualized treatment, sarcoma, squamous carcinoma, staging, vaginal cancer

## Abstract

Diagnosis of a primary vaginal cancer is rare, as most vaginal tumors are metastatic from another primary site. Although cancer of the vagina is more common in postmenopausal women, an increase in young women being diagnosed with primary vaginal cancer has been reported, especially in countries with a high HIV prevalence. This is associated with persistence of high‐risk HPV infection. The emphasis should be on primary prevention with prophylactic HPV vaccination. Once there is a suspicion of a primary vaginal cancer, this should be confirmed histologically with biopsy. Staging has been done clinically, as with cervical cancer; however, there is a role for imaging in assisting with staging as this is often a difficult assessment. Treatment should be individualized and depends on stage as well as histologic subtype. It is prudent to refer cases to centers of excellence with experience in dealing with this rare gynecological cancer.

## Synopsis

A review of the different histologic subtypes of primary vaginal cancer in terms of staging and role of imaging, as well as treatment options and prognoses.

## 1 INTRODUCTION

Primary vaginal cancer is rare, constituting only 1%–2% of all female genital tract malignancies and only 10% of all vaginal malignant neoplasms.^1^ It is strictly defined as a cancer found in the vagina without clinical or histologic evidence of cervical or vulvar cancer, or a prior history of these cancers within 5 years.^2^ To support this, most suspicious lesions of malignancy in the vagina will correspond to metastatic lesions of cervical or vulvar cancer, or others metastasizing to the vagina (e.g. breast, endometrium, trophoblast, ovary, lymphoma).

Historically these cancers are more common in elderly and postmenopausal women. If vaginal malignancy is found in younger women, it is usually etiologically linked to cervical cancer, specifically with regard to the persistence of high‐risk HPV infections.^3^ Vaginal cancers, although rare, are increasingly being seen in younger women owing to the increase in persistent high‐risk HPV infections, especially in settings with a high HIV prevalence.

## 2 ANATOMY

The vagina is an elastic muscular tube comprising many mucosal folds. It extends from the cervix of the uterus to the hymenal ring and lies posterior to the bladder and anterior to the rectum. Its elasticity and dimensions vary depending on a woman's age, parity, previous surgeries, and hormonal status. Such characteristics can make proper examination (limited by pain or narrowness of the introitus) and identification of small malignant lesions difficult.

## 3 PREVENTION

### 3.1 Primary prevention (vaccination)

As with premalignant cervical lesions and carcinoma of the cervix, persistent HPV infection—particularly the HPV 16 subtype—has been associated with the long‐term development of high‐grade squamous intraepithelial lesion (HSIL) and carcinoma of the vagina.^4^, ^5^, ^6^ The introduction of HPV vaccination as a primary prevention strategy in cervical cancer has also been shown to reduce the prevalence of noncervical premalignant lesions among vaccinated women.^7^ Long‐term trend analyses from the Norwegian Cancer Register also show promising estimates of reduction in HPV‐associated cases of vaginal cancer in future years among HPV‐vaccinated communities.^8^

### 3.2 Secondary prevention (screening)

There is no evidence to indicate routine screening for vaginal cancer after hysterectomy for benign disease. If a hysterectomy has been performed for persistent HSIL after repeated excisional procedures of the cervix, vault smears and colposcopy are recommended for long‐term follow‐up.^9^, ^10^

In those cases where screening for vaginal cancer is indicated, HPV tests adequately correlate with cytological findings.^11^ Independently of that, co‐testing seems to offer better accuracy in the diagnosis of recurrent disease—as observed during follow‐up after treatment of premalignant cervical lesions.^12^

### 3.3 Tertiary prevention (management of precancerous lesions)

As the majority of vaginal cancers are of squamous histology, a common etiology is shared with cervical cancer. This is the persistence of high‐risk/oncogenic HPV infections. Co‐factors include immunosuppression and cigarette smoking. Smoking in combination with high‐risk HPV increases the risk of progression to vaginal HSIL when compared with nonsmokers.^13^

The terminology for precancerous HPV‐associated vaginal lesions has changed over time. In 2014, the WHO classification replaced the three‐tiered classification used previously (VAIN 1–3) with squamous intraepithelial lesion (SIL). This is divided into two categories: low grade (LSIL) and high grade (HSIL). LSIL (VAIN 1) may be associated with either low‐risk or high‐risk HPV and it represents productive or transient infections that may regress. In contrast, HSIL represents transforming high‐risk infections (previously VAIN 2–3).^14^

Women with HSIL are usually asymptomatic and most women are aged over 60 years. HSIL can be seen in younger women, especially in immunocompromised individuals (HIV and transplant patients). Colposcopy with acetic acid and /or Lugol iodine is indicated if a woman has an abnormal vaginal cytological smear and no gross abnormality. Biopsies of colposcopically abnormal areas (acetowhite areas with punctation and/or punctation and mosaicism) are essential for the diagnosis.

Risk of progression of HSIL to invasive cancer has been found to range between 2% and 12%.^15^, ^16^

Treatment of precancerous lesions of the vagina must be individualized. Biopsy‐proven LSIL lesions can be followed up with observation only (repeat smears and colposcopy), especially if women have non‐oncogenic strains of HPV.

The various modalities of treatment for HSIL lesions include laser ablation, surgical excision, and topical treatments such as imiquimod and topical chemotherapy with 5‐fluorouracil (5FU). Choice of treatment depends on the number and location of the lesions, and the degree of suspicion for an invasive cancer, as well as the availability of various treatment options, cost of treatment, and skill of the treating doctor.

#### 3.3.1 Surgical excision

The advantages of excising HSIL of the vagina is that it provides a pathological description of the tissue removed, especially when there is a suspicion of microinvasion on colposcopic evaluation. Most HSIL lesions are in the vault or upper third of the vagina and excision is ideal in this situation.^17^ Morbidity can be associated with the proximity of structures such as the bladder and rectum, as well as associated dyspareunia as a result of vaginal shortening and stenosis. Women should be extensively counselled preoperatively.

#### 3.3.2 CO_2_ laser vaporization

Laser vaporization is an alternative option to excision, especially for multifocal lesions. Since there is no histologic specimen with this procedure, it is imperative to ensure that there is no concern regarding cancer.^18^, ^19^

#### 3.3.3 *Topical 5*‐*fluorouracil*

Topical application of 5FU has been used in an effort to avoid the mutilating adverse effects of excision and laser ablation. Older studies have shown comparable efficacy to laser and excision.^20^, ^21^ However, these are retrospective in nature with small numbers. More recent studies have shown inferior efficacy.^15^, ^22^ A 2018 retrospective study reviewed recurrence rate in 576 women treated with topical 5FU, excision, or laser ablation. The statistically significant factors that contributed to recurrence and progression were the presence of high‐risk HPV positivity and treatment modality. Laser and excision were found to be superior to topical management with 5FU in reducing recurrences. It was also found that laser was more effective than excision in reducing recurrences of multifocal lesions, whereas surgical excision was preferable with unifocal HSIL.^23^

#### 3.3.4 Topical 5% imiquimod

Imiquimod (applied topically either in creams or drug‐embedded tampons) is an immune response modulator that activates the innate immune response and subsequently induces proinflammatory factors such as interferons. It provides an alternative noninvasive and safe method for treating women with vaginal HSIL, especially young women with multifocal lesions or older women who wish to avoid surgical modalities. Studies on imiquimod are few, but a 2016 randomized controlled trial (RCT) as well as a systematic review and meta‐analysis (including this RCT) in 2017 found that this is a safe and effective treatment with regression rates similar to laser. HPV clearance rates were greater than 50%, which was superior to laser.^24^, ^25^

In general, independent of therapy chosen, most patients require more than one therapy for adequate control. Since recurrence rate is high with any single therapy, close and extended follow‐up is recommended in all cases. As in treated premalignant cervical lesions, both HPV testing and cytology are recommended.

## 4 MANAGEMENT OF INVASIVE CANCER OF THE VAGINA

Women usually present with bleeding or a malodorous discharge. Locally advanced disease may also result in pain, and symptoms of disseminated disease depend on the site of metastases.

Diagnosis of vaginal cancer is made with directed biopsy of the lesion and a clinical assessment that ensures there is no evidence of tumor on the cervix or the vulva. Biopsy can be performed in an outpatient setting; however, sometimes examination under anesthesia is required for diagnosis and adequate assessment, particularly in young girls and elderly women where the exam can become painful or limited by narrowness of the introitus.

As primary vaginal cancer is rare, treatment is complex, often individualized, and much of the management is extrapolated from cervical cancer owing to its similar etiology and anatomical location. Therefore, it is recommended that women diagnosed with primary vaginal cancer are referred to a tertiary unit whenever possible. Accordingly, outcomes are influenced by the experience of the medical team in treating this type of rare tumor.^26^

## 5 PATTERNS OF SPREAD

### 5.1 Direct extension

A vaginal tumor may extend to the surrounding pelvic soft tissue structures, including paravaginal tissue, parametria, urethra, bladder, and rectum. Most tumors occur in the upper third of the vagina, especially the posterior wall.

### 5.2 Lymphatic spread

The lymphatic drainage of the vagina is complex. The upper vagina drains by lymphatics to the pelvic lymph nodes. This includes the obturator, internal iliac (hypogastric), and external iliac nodes. Spread to the para‐aortic lymph nodes is rare. The lower vagina drains to the inguinal and femoral nodes (groin nodes). Cancer in the midvagina may follow both pelvic and groin node routes.^27^

### 5.3 Hematogenous spread

Hematogenous dissemination to lung, liver, and bone are usually late manifestations.

## 6 HISTOLOGIC SUBTYPES

The predominant histologic subtype in primary vaginal cancer is squamous carcinoma, which comprises 90% of cases. Adenocarcinoma accounts for about 8%–10% of cases. Lymphomas, sarcomas, and melanomas of the vagina are extremely rare.

All malignancies must be confirmed histologically. Since 80% of cases are metastatic or secondary tumors, it is important to ensure that there is no other primary site by careful examination and appropriate investigations. Although biopsy can be obtained under local anesthesia, in many instances examination under sedation or general anesthesia should be performed to undertake an adequate exam and obtain a biopsy.

By definition, tumors in the vagina that touch or extend to the external os of the cervix should be classified as cervical cancer.

## 7 GRADING

- GX: Grade cannot be assessed.
- G1: Well differentiated.
- G2: Moderately differentiated.
- G3: Poorly or undifferentiated.

## 8 STAGING

Vaginal carcinoma is primarily clinically staged. This is based on the results of a physical exam, biopsy, and imaging tests performed before treatment selection. The 2009 FIGO staging for primary vaginal cancer is described and compared with other systems in Table 1.

**TABLE 1:** Comparison of staging systems for vaginal cancer

AJCC Stage	Stage grouping (TNM)	FIGO Stage	Stage description
IA	T1a N0 M0	I	The cancer is only in the vagina and is no larger than 2.0 cm (4/5 inch) (T1a) It has not spread to nearby lymph nodes (N0) or to distant sites (M0)
IB	T1b N0 M0	I	The cancer is only in the vagina and is larger than 2.0 cm (4/5 inch) (T1b) It has not spread to nearby lymph nodes (N0) or to distant sites (M0
IIA	T2a N0 M0	II	The cancer has grown through the vaginal wall, but not as far as the pelvic wall and is no larger than 2.0 cm (4/5 inch) (T2a) It has not spread to nearby lymph nodes (N0) or to distant sites (M0)
IIB	T2b N0 M0	II	The cancer has grown through the vaginal wall, but not as far as the pelvic wall and is larger than 2.0 cm (4/5 inch) (T2b) It has not spread to nearby lymph nodes (N0) or to distant sites (M0)
III	T1 to T3 N1 M0	III	The cancer can be any size and might be growing into the pelvic wall, and/or growing into the lower one‐third of the vagina and/or has blocked the flow of urine (hydronephrosis), which is causing kidney problems (T1 to T3). It has also spread to nearby lymph nodes in the pelvis or groin (inguinal) area (N1) but not distant sites (M0)
	OR		
	T3 N0 M0	III	The cancer is growing into the pelvic wall, and/or growing into the lower one‐third of the vagina and/or has blocked the flow of urine (hydronephrosis), which is causing kidney problems (T3) It has not spread to nearby lymph nodes (N0) or to distant sites (M0)
IVA	T4 Any N M0	IVA	The cancer is growing into the bladder or rectum or is growing out of the pelvis (T4) It might or might not have spread to lymph nodes in the pelvis or groin (inguinal area) (Any N). It has not spread to distant sites (M0)
IVB	Any T Any N M1	IVB	The cancer has spread to distant organs such as the lungs or bones (M1). It can be any size and might or might not have grown into nearby structures or organs (Any T) It might or might not have spread to nearby lymph nodes (Any N)

## 9 ROLE OF IMAGING TECHNIQUES IN DIAGNOSIS, STAGING, AND TREATMENT

Vaginal cancer is currently staged clinically, as is cervical cancer. Modern imaging techniques such as computed tomography (CT), magnetic resonance imaging (MRI), and positron emission tomography (PET) are encouraged to guide management; however, these tests should not be used to change the initial clinical staging.^28^ In this regard, the FIGO Gynecologic Oncology Committee recommends that wherever available, imaging should be used to better define tumor volume and extension of disease. The Committee also encourages registering of imaging findings and their influence on therapeutic decisions as a complement of assigned clinical FIGO stage. This will allow further analyses that could change the current staging system.

### 9.1 Magnetic resonance imaging

As extrapolated from cervical cancer, MRI is more sensitive in detecting tumor size, as well as paravaginal or parametrial involvement.^29^, ^30^ In primary vaginal tumors, clinical assessment may be difficult and MRI may be a useful tool in individual cases owing to its superior soft tissue resolution.^26^

### 9.2 PET‐CT

As in cervical cancer, the use of PET‐CT in primary vaginal cancer has been found to be superior compared with other imaging modalities for detecting nodal disease.^31^ It is also useful in detecting recurrent disease.

## 10 TREATMENT

The treatment of carcinoma of the vagina depends primarily on histology, tumor volume, anatomical localization of the lesion, stage of the disease, and age of the patient. Adversely, owing to anatomical localization, both reproductive potential (young women) and sexual function (any age) can be affected. Different modalities of treatment can be offered to patients affected by this disease including surgery, radiotherapy, chemotherapy, or a combination of some or all of the above.

### 10.1 Surgery

The role of surgery is limited in primary vaginal cancer since the primary tumor is in close proximity to the bladder, urethra, and rectum. In general, primary treatment with surgery is limited to early and small lesions confined to the vaginal mucosa (less than 2 cm). There are no RCTs and most of the literature is retrospective in nature. There may be a role for surgical treatment in certain additional situations beyond early disease, as listed below.

1. Surgical management of Stage I disease (early disease)
  - Upper vaginal disease
    - The tumor is limited to the mucosa in Stage I disease.
    - If the uterus is in situ, radical hysterectomy, vaginectomy aiming for 1 cm disease‐free margins, and pelvic lymphadenopathy should be offered. If the uterus has been removed, radical vaginectomy as above with pelvic lymphadenopathy can be performed.^32^, ^33^
  - Lower vaginal disease
    - Radical wide local excision with 1 cm margins can be offered, in addition to bilateral groin node dissection.
2. Ovarian transposition during surgery and preradiation

In young women with vaginal cancer requiring radiation as primary treatment, ovarian transposition can be offered prior to definitive radiation treatment in an effort to prevent the adverse effects of radiation‐induced menopause. In selected cases, laparoscopic or extraperitoneal removal of bulky lymph nodes can be offered as part of staging and treatment planning.

- Central recurrence after radiation treatment

Pelvic exenteration is a possibility if the recurrence is central and isolated. These patients require extensive counselling regarding the risks and morbidity of surgery, as well as the impact on quality of life and body image.

- Palliative management of recurrent or advanced disease

In women with advanced (Stage IV disease) or recurrent disease who present with vesicovaginal or rectovaginal fistula, a palliative urinary diversion or colostomy can be offered to improve quality of life before definitive management with radiation treatment.

### 10.2 Radiation

In the majority of cases and especially in advanced stages, radiation constitutes the cornerstone of treatment for this disease. This is a combination of external beam radiation (EBRT) and intracavitary radiotherapy or brachytherapy (ICRT). The principal advantage of radiation is organ preservation.

As in other pelvic malignancies, MRI has become an essential component for properly defining tumor volume and the spatial relationship of the tumor with neighboring organs during treatment planning.

EBRT to the pelvis includes the external iliac and obturator nodes as per standard of care. In addition, the inguinal nodes may be included if the tumor is in the distal vagina. Brachytherapy is used to deliver a boost of high‐dose radiation to the residual tumor, and the use of brachytherapy is associated with longer overall survival.^34^ Image‐guided or adaptive brachytherapy with CT or MRI offers the potential to give higher doses of radiation to the residual tumor and decrease radiation dose to surrounding healthy organs.^35^

The optimal or lower threshold dose is 70 Gy, which has been shown to improve outcomes.^36^, ^37^, ^38^ Doses higher than 70 Gy result in significant grade 3 and 4 toxicities.^39^

Intensity modulated radiation therapy (IMRT) is an advanced form of radiation that allows for higher dosages of radiation to be delivered to the cancer. Although studies in vaginal cancer are limited, this form of radiation may allow improved dosages to the cancer, with fewer adverse effects because dose to the adjacent structures is limited.^40^

### 10.3 Concurrent chemotherapy with radiation

Modern management of vaginal cancer often combines concurrent chemotherapy such as cisplatin or 5FU. This has been extrapolated from the successful outcomes of this treatment with cervical cancer.^40^ However, most studies using chemoradiation in vaginal cancer are limited owing to the small numbers of cases and lack of comparison to radiation on its own.^41^, ^42^ Chemoradiation may be considered in the treatment of vaginal cancer following a 2013 retrospective review suggesting a potential improvement in overall and disease‐free survival.^43^ Although this was a small review (71 patients), it showed a significant difference between both overall survival and disease‐free survival when comparing women who received radiation alone versus chemoradiation as primary treatment (three‐year overall survival of 56% versus 79% and three‐year disease‐free survival of 43% versus 73%).^43^

The combination of EBRT and brachytherapy to a total dose of 70–80 Gy with concomitant weekly cisplatin‐based chemotherapy is the current standard of care for locally advanced vaginal cancer.^42^

## 11 SPECIAL SITUATIONS

### 11.1 Adenocarcinoma of the vagina

Primary adenocarcinoma of the vagina accounts for 8%–10% of cases. In 1971, Herbst et al.^44^ described the association between diethylstilbestrol (DES) and clear cell adenocarcinoma (cervix/vagina) in daughters who had intrauterine exposure during the first 16 weeks of pregnancy. Most of these cases were diagnosed between the ages of 14 and 22 years. A 2017 retrospective review of 420 cases found that most cases were in younger women, but there was a small second peak at 42 years.^45^ Intrauterine DES has been banned since the 1970s and it is expected, therefore, that clear cell adenocarcinomas attributable to intrauterine DES exposure will disappear in the next decades. For women in doubt or certain of intrauterine exposure to DES, closer follow‐up is recommended including annual cytology (the so‐called four‐quadrant Pap test, with extensive sampling of the cervix and vagina) and careful visualization of both the cervix and vagina (including colposcopy and Lugol staining whenever there is a clinical suspicion).^46^, ^47^ In addition, the so‐called “DES daughters” should be screened closely with mammography for the slight increase in risk of developing breast cancer.^48^

Non‐DES adenocarcinoma is exceptionally rare and behaves as a different entity with a different natural history. This may include endometrioid (arising from endometriosis) or mucinous subtypes, which are typically diagnosed in postmenopausal women.

#### 11.1.1 Treatment

Most adenocarcinomas are treated in the same manner as squamous carcinomas.

### 11.2 Vaginal melanoma

Vaginal melanoma is exceptionally rare and is typically diagnosed in elderly women. The incidence is approximately 3 per 10 million women per year.^49^

#### 11.2.1 Treatment

There is no standard of treatment in the literature owing to its rarity, but surgical excision either by wide local excision or colpectomy with or without pelvic exenteration has been described.^50^, ^51^ Adjuvant treatment in the form of radiation or immunotherapy (interferon‐alpha) can be considered, although there is limited evidence to support this. Palliative chemotherapy or radiation can be used in advanced disease.^52^

### 11.3 Sarcoma botryoides

Rhabdomyosarcomas are the most common soft tissue cancers in children and adolescents, accounting for 4%–6% of all malignancies in this age group. Twenty percent of these occur in the lower genital tract, and more than 50% are of the embryonal histologic subtype.^53^, ^54^

The 2013 WHO classification of tumors of soft tissue and bone stratified rhabdomyosarcomas into four main histologic subtypes^55^:

- Typical embryonal variant (most common accounting for 58% of cases): this comprises the classic botryoides.
- Spindle cell/sclerosing variant.
- Alveolar variant.
- Pleomorphic variant.

Most rhabdomyosarcomas in children are in the vagina, whereas adolescents have predominantly cervical lesions. Sarcoma botryoides often presents in the first few years of life with bleeding and nodular lesions filling and possibly protruding from the vagina (grape‐like). More advanced stages of disease may present with abdominal pain, an abdominal mass, or symptoms of distant metastases.

#### 11.3.1 Treatment

These tumors are rare and, as a result, treatment is based on the case reports and series that have been collected over time. There is no level 1 evidence to support best treatment options. It is recommended that a multidisciplinary team be involved in decisions regarding treatment, especially when this involves children and adolescents. Ideally these patients should be referred to centers of excellence where there has been appropriate experience in dealing with these cases.

Treatment previously involved extensive surgery, but a series of case reports has suggested that less radical surgery can be done. These reports have shown good outcomes in terms of survival as well as quality of life if the patients are selected carefully. Unfavorable factors such as the cervix as the primary site, large lesion size, and extent of the disease may require radical surgical intervention or chemotherapy. In the absence of these factors, wide local excision and chemotherapy have been used with good success rates. Neoadjuvant chemotherapy is another primary choice that can be used, followed by surgical resection. Radiotherapy should be avoided, if possible, as there are long‐term adverse effects.

## 12 PROGNOSES AND OUTCOMES OF VAGINAL CANCER

The main determinant of prognoses in carcinoma of the vagina is the stage of disease at the time of diagnosis, independently of subjacent histology. Additional factors influencing prognosis in squamous subtype are the tumor volume (>4 cm), its location outside of the upper third of the vagina, HPV status, and MIB‐1 index.^56^ As with other malignancies, age, reproductive and sexual functions, and performance status can influence the selection of specific therapies and potentially affect survival outcomes. However, the non‐negotiable premise is to offer the best option in terms of survival. When comparing histologies, the better outcome is obtained in squamous carcinoma of the vagina when diagnosed at earlier stages (I or II). In the last three decades, incorporation of imaging techniques during diagnosis and treatment planning has influenced decision‐making and the selection of treatment for cases with similar clinical stage but with different tumor volume. As in cervical cancer, the addition of concurrent chemotherapy to radiotherapy has also improved survival in the squamous histology.

Since vaginal carcinoma is a rare entity, most of the available data in terms of therapeutic outcomes come from single institutions and include cohorts of patients from different epochs where different treatment modalities were used. For example, one of the largest studies published by the MD Anderson Cancer Center includes193 patients treated over two decades. This study reported five‐year disease‐specific survival rates of 85% for 50 patients with Stage I disease, 78% for 97 patients with Stage II, and 58% for 46 patients with Stages III–IVA.^57^ A 2013 retrospective analysis from the Department of Radiation Oncology at Stanford University, also including a long experience over five decades, reflects the positive impact of incorporating new imaging technologies (e.g. MRI and PET‐CT) in defining primary lesion characteristics, unveiling hidden metastatic disease, and influencing treatment planning. Adding imaging techniques no doubt influences decision‐making, favoring the use of chemoradiation and IMRT, and results in the improvement of locoregional control rate, and distant metastasis‐free and overall survival. This is particularly useful in the management of large volume tumors (particularly ≥4 cm) minimizing grade 3–4 toxicities.^39^ Confirming such an improvement, a 2015 review from Gadducci et al.^56^ summarized the data available and obtained a five‐year overall survival that varies between 35% and 78%, with a severe late complication rate between 9.4% and 23.1% among cases of squamous carcinoma of the vagina when treated with different standards of radiotherapy.

In terms of prognoses for other histologies, young women with early‐stage DES‐related adenocarcinomas treated with either radiation, surgery, or a multimodal approach have good outcomes with reported five‐year survival of 80%–87%.^58^, ^59^ This is in contrast to non‐DES‐related adenocarcinomas, which have a worse prognosis owing to an increased risk of both local and distant metastases. A review of 26 women with non‐DES‐related vaginal cancer at the MD Anderson Cancer Center found an overall five‐year survival of 34%.^60^

Vaginal melanoma has a very poor prognosis, with a five‐year overall survival of 15%.^51^ In a 2017 systematic review of 805 cases reported over the last 20 years (mostly case reports), the mean recurrence‐free survival was short, at 16 months, with a mean overall survival of only 22 months.^52^

Finally, in relation to sarcoma botryoides, most of the available information in the literature comes from the reports of small series from single institutions (many of them combining different variants including the embryonal or botryoides subtype and from different origins in the genital tract). A 2017 report from Peking Union Medical College Hospital, including eight rhabdomyosarcoma cases of the female genital tract over a 20‐year period, showed that after combined therapy with local excision and chemotherapy, most patients can achieve a good prognosis. The prognosis is highly correlated with tumor site and histologic type.^61^ Similarly, in a 2017 survey of 144 eligible cases of rhabdomyosarcomas of the lower female genital tract in the SEER database (between 1973 and 2013)—75.7% corresponding to the embryonal subtype—the estimated five‐year overall survival rate was 68.4%. Among the factors associated with better overall survival were younger age, absence of distant metastasis, embryonal histology, negative lymph nodes, and the performance of surgery. An important conclusion of this survey was that for prepubescent girls and adolescents, the use of radical surgery did not confer a survival benefit compared with local tumor excision.^62^

## AUTHOR CONTRIBUTIONS

TA and MC shared the concept design, literature review, and writing of the 2018 manuscript, which was updated by LR and TA in 2021.

## CONFLICT OF INTEREST

The authors have no conflicts of interest to declare.
